# Children’s and Their Parents’ Experiences With Home-Based Guided Hypnotherapy: Qualitative Study

**DOI:** 10.2196/58301

**Published:** 2025-01-27

**Authors:** Ilse N Ganzevoort, Adriëlla L van der Veen, Manna A Alma, Marjolein Y Berger, Gea A Holtman

**Affiliations:** 1 Department of Primary- and Long-term Care, University Medical Center Groningen, University of Groningen, 9700 AD, Oostersingel, Groningen, PO-box 196, Netherlands, 31 653445206; 2 Department of Health Sciences, University Medical Center Groningen, University of Groningen, Groningen, Netherlands

**Keywords:** qualitative study, primary health care, children, functional abdominal pain, irritable bowel syndrome, hypnotherapy, eHealth, abdominal pain, child, parents, accessibility, questionnaire, interviews, thematic analysis, home guided, primary care, mobile phone

## Abstract

**Background:**

Management of children with functional abdominal pain (FAP) or irritable bowel syndrome (IBS) is difficult in primary care. When education and reassurance do not alleviate symptoms, primary care physicians lack treatment options for children with FAP or IBS. Home-based guided hypnotherapy is a promising treatment because of its accessibility. To address feasibility, it is of utmost importance to take experiences from children and their parents into account.

**Objective:**

We aimed to explore children’s and their parents’ experiences with home-based guided hypnotherapy for children with FAP or IBS.

**Methods:**

This qualitative study used open-ended questions from a questionnaire and in-depth semistructured interviews with children and their parents who had a hypnotherapy intervention prescribed. The interviews were audio-recorded and transcribed verbatim. Data were collected and analyzed iteratively using thematic content analysis.

**Results:**

A total of 76 children were eligible, and we collected questionnaire data from 56 children. A total of 23 interviews were conducted with 10 children and 15 parents. Six themes emerged from questionnaire data and interviews: impression of the exercises, not for everyone, influence of perceived effect, integrating exercises in daily life, content and practicalities of the website, and customization to personal preferences. Children with FAP or IBS experienced home-based guided hypnotherapy and the exercises differently, ranging from boring to fun. From interviews with the parents, it emerged that hypnotherapy is not suitable for everyone; for example, when children are very young or have a low developmental level, cannot sit still, cannot surrender to the exercises, or are too energetic or stressed, it might be difficult to comply. Experiences were shaped by the influence of a perceived effect and to which extent children were able to integrate exercises in daily life. The content and practicalities of the website also influenced experiences, and hypnotherapy that is adaptable to personal preferences, including by appearance and content, would be highly appreciated.

**Conclusions:**

The children and parents experienced home-based guided hypnotherapy differently, ranging from boring to fun. Hypnotherapy might be difficult or boring for some children. The children enjoyed hypnotherapy when they liked the topic or story, felt positive effects, could easily integrate exercises in daily life, or enjoyed the website in general. The children’s experiences and adherence can be further improved by adding short exercises and customizing hypnotherapy to their personal preferences on the website’s appearance and content. This could increase effectiveness but must be studied further.

## Introduction

Disorders of gut-brain interaction such as functional abdominal pain (FAP) and irritable bowel syndrome (IBS) are chronic pain conditions without organic cause [1]. These disorders are common in primary care, as a general practitioner (GP) sees approximately 10 children with disorders of gut-brain interaction each year [2,3]. Dutch GPs diagnose FAP or IBS when after medical history and physical examination, no underlying tissue damage, somatic causes, or metabolic or anatomic abnormalities can explain the symptoms of the child [4]. FAP and IBS are associated with lower quality of life, school absence, and higher anxiety and depression scores [5,6], and around 50% of children in primary care still report abdominal complaints one year later [7]. Management consists of education and reassurance, but if this fails to alleviate symptoms, there are few evidence-based treatment options in primary care. Children with abdominal pain and their parents report a desire for receiving a specific diagnosis and a need for information about its cause and treatment options [8-10]. Children often adopt coping mechanisms by themselves, such as reassurance and a calm approach, distraction techniques, breathing exercises, and bedtime meditation [11]. Hypnotherapy could be a treatment option, as it has shown to be effective in children referred to secondary pediatric care [12,13]. However, it has not been studied in primary care. Research in primary care is important because a different setting, selection of patients, and organization of care might influence treatment effects.

With hypnotherapy, a patient is induced into a hypnotic state and guided to suggestions by a therapist or by listening to audio-recorded exercises in their home environment (ie, home-based guided hypnotherapy) [13,14]. Home-based guided hypnotherapy is promising in primary care because of its accessibility [15]. Very few mild to moderate side effects of hypnotherapy are reported [16], and the fact that children can do it by themselves without involvement of others makes primary care an interesting setting. Experiences with home-based guided hypnotherapy in children with FAP or IBS have not been studied yet. Insights in experiences of children and their parents are important for successful implementation [17]. In this study, we aimed to explore experiences of children with FAP or IBS and their parents with home-based guided hypnotherapy, and to capture their ideas about potential areas for improvement.

## Methods

### Design

This qualitative study is part of the ZelfHy study, a randomized controlled trial (RCT) evaluating the effectiveness and cost-effectiveness of home-based guided hypnotherapy in children with FAP or IBS in primary care (ClinicalTrials.gov NCT05636358) [18]. The participants in the intervention group had access to a 3-month, home-based, guided hypnotherapy program. This study used questionnaire data of open questions and log data from the ZelfHy study of participants who received the intervention, and in-depth, semistructured interviews with a purposively selected sample of children and their parents.

We followed the Consolidated Criteria for Reporting Qualitative Research [19].

### Home-Based Guided Hypnotherapy Program

**Figure 1.**
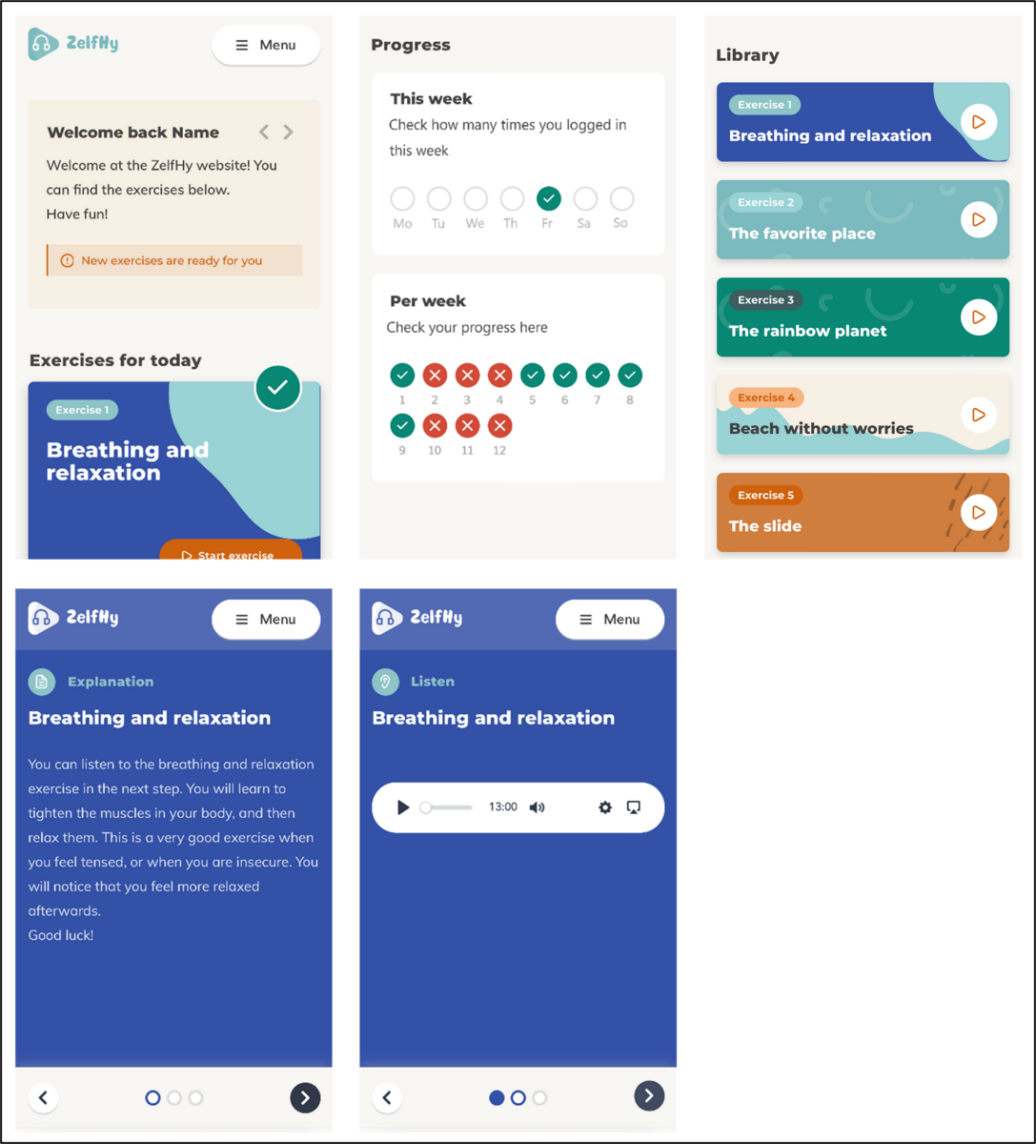
Screenshots of the website taken from a smartphone.

The children in the intervention group of the RCT received standard care and home-based guided hypnotherapy for 3 months. Before starting, a researcher explained hypnotherapy, its benefits for abdominal pain, and access methods during a video call with the child, parent, or both. They were also advised not to discuss the pain. The hypnotherapy program included 5 exercises: 1 breathing and relaxation exercise and 4 visualization exercises: “the favorite place” or “the favorite place+ rainbow” (age-dependent), “the rainbow planet” or “air balloon” (age-dependent), “the beach without worries,” and “the slide.” Two exercises comprised 2 versions with adjustments in language: for children aged <12 years and ≥12 years. Exercises were audio-recorded by a hypnotherapist. Instructions and exercises were hosted on a responsive website, as shown in Figure 1. All exercises were immediately available, but the children were guided to listen to the first 2 exercises for the first 2 weeks, adding new exercises every 1 to 2 weeks. They could choose and repeat exercises whenever they wanted. The children were encouraged to practice at least five times a week for 15‐20 minutes daily for over 3 months. Automatic email reminders were sent after 14 and 28 days of inactivity to improve compliance.

### Patient Participation

One mother of a girl aged 9 years and one male adolescent aged 19 years who both finished the intervention were engaged, and both received a voucher. Patient participation included attending 2 research team meetings and member checking the final results. The 2 research team meetings included discussion of the interview guide before the interviews, and discussion of topics, codes, and preliminary themes after the first interviews.

### Recruitment and Participants

In this qualitative study, all the children and their parents in the intervention group of the RCT were included. Children aged 7‐17 years with FAP or IBS according to their GP participated in the RCT. Exclusion criteria for participation in the RCT were a concomitant organic gastrointestinal disease, being managed for abdominal pain by a pediatrician, intellectual disability, psychotic disorders, a history of hypnotherapy in the past year, and poor comprehension of the Dutch language. Detailed information of recruitment methods for the ZelfHy study are described elsewhere [18].

All the children and their parents were invited to answer the questionnaire. Additional inclusion criteria for in-depth interviews for this study were that the children had to be within the 3-month intervention time frame at that time, and live in the Northern or Middle part of the Netherlands because of travel convenience. Purposive sampling was used to achieve diversity in age, gender, and therapy adherence based on automatically logged data for the user interactions. The first author, a female primary care researcher, invited parent-child dyads for interviews via telephone approximately 6 weeks after they started the intervention. We assumed that halfway through the intervention, the children listened to all exercises and were able to recall and express their experiences. In practice, with time delay such as decision to participate and setting a date for the interview, interviews would be held in weeks 8‐12. The authors believe that this was enough time to shape experiences.

### Data Collection

Questionnaire data consisted of open-ended questions regarding what they liked and disliked generally, and for each exercise separately (Multimedia Appendix 1). Questionnaire data were collected through an electronic data capture system at 3 months follow-up between March 2021 and January 2024. The parents completed the questionnaire for the children aged <12 years, and the children aged ≥12 years completed the questionnaire themselves, with parental help as needed. Additionally, we collected log data from the intervention consisting of how long the children listened to each exercise per session. A session was defined as a log-in and start of at least one exercise. In accordance with a previous study on the effectiveness of hypnotherapy, we defined adequate use as starting at least four different exercises [18,20].

From May 2023 to December 2023, a female primary care researcher and trained interviewer (ING) and a female social scientist with expertise in qualitative research and trained interviewer (MAA) conducted semistructured interviews. The first author had contact via telephone with the parents for study procedures for the ZelfHy study before the interview. The interviews took place at the participant’s home, except for the children with low adherence. For convenience, the parents of these children were interviewed by telephone and they did not necessarily have to live in the Northern or Middle part of the Netherlands. Interviews were recorded using a digital voice recorder and transcribed verbatim. Field notes and short memos were written during and after each interview.

To develop the semistructured interview guide, we used sensitizing concepts from literature and expert discussion with the research team which consisted of a female primary care researcher, a female primary care research assistant, one female epidemiologist, one female social scientist, and two female GPs. The interview guide consisted of open-ended questions about the children’s and parents’ experiences with the therapy (Multimedia Appendix 2). Interviews were performed until data saturation was reached (ie, interviews no longer generated relevant concepts). We completed 3 additional interviews with the children and their parents in which no new codes were found. Iterative meetings with the research group were held to evaluate and update the interview guide for new concepts and discuss data saturation.

### Data Analysis

Thematic content analysis was conducted as proposed by Braun and Clarke for questionnaire data and interview transcripts [21]. First, all questionnaire data and 10 interview transcripts were read and inductively and independently coded by ING (both questionnaire data and interview transcripts), GAH (questionnaire data and female epidemiologist), and ALvdV (interview transcripts and female primary care research assistant). ING coded the remaining transcripts and ALvdV checked the coding of these transcripts. Inconsistencies between coders were discussed until consensus was reached. Consequently, emerging themes and subthemes were discussed and redefined with the research team until consensus was reached. In one of these meetings, a mother and adolescent were part of the research team. Illustrative quotes were translated from Dutch to English by a native English speaker and editor, and the first author checked whether their meaning was retained. All analyses were facilitated using Atlas.ti (version 23; ATLAS.ti Scientific Software Development GmbH) software.

### Ethical Considerations

The Medical Ethics Review Committee of the University Medical Center Groningen, the Netherlands, confirmed that the Medical Research Involving Human Subjects Act which includes the Declaration of Helsinki, did not apply to this qualitative study (number 202200110). All the participants gave informed consent. Participant data were deidentified after the interviews. No compensation was provided.

## Results

### Participants

**Figure 2.**
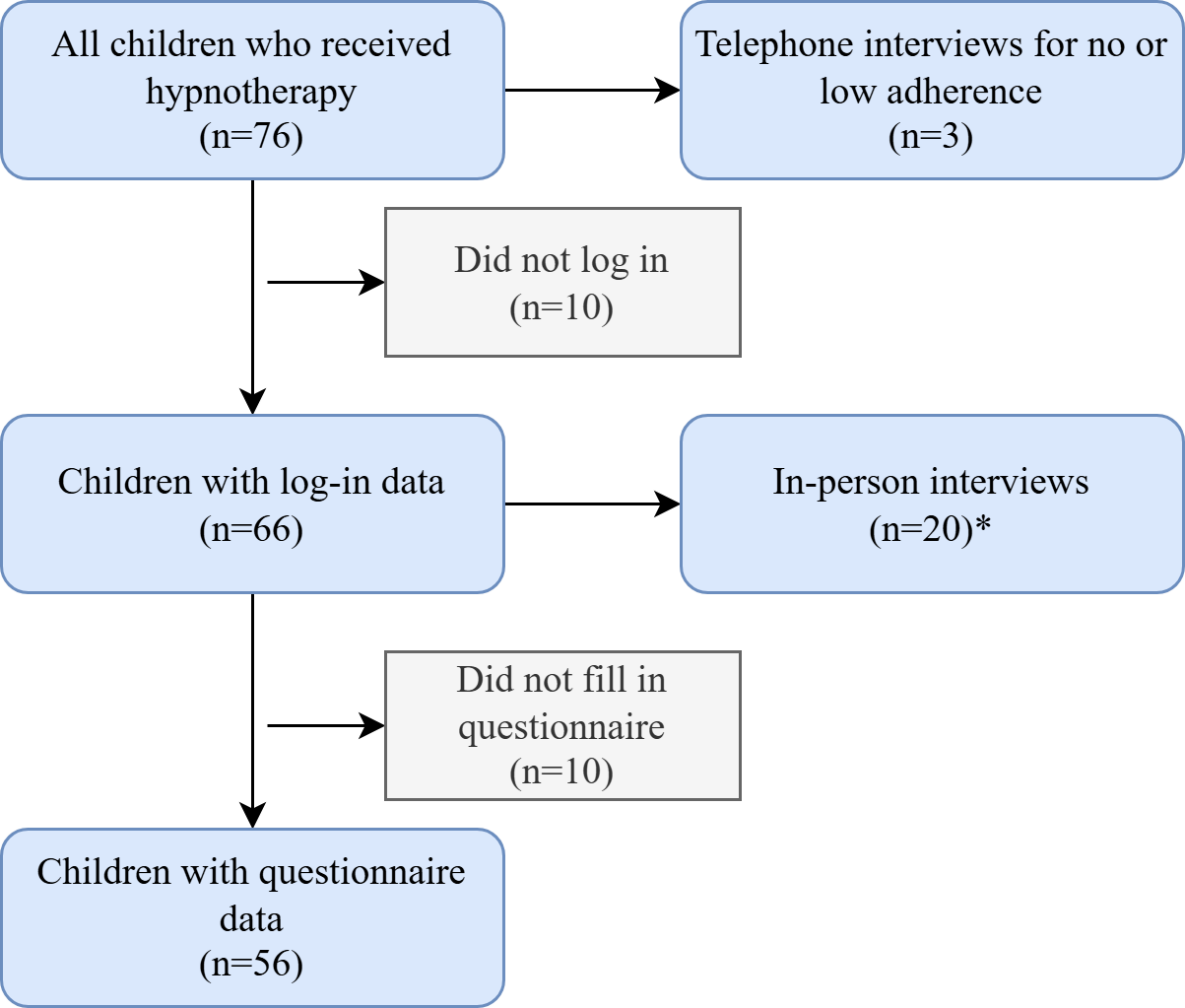
Flowchart of participants. Asterisk (*) denotes that 20 in-person interviews were conducted: 10 with children and 10 with parents.

In total, 76 children were eligible from the intervention group of the RCT. Their median age was 9.1 (IQR 8.1‐11.2) years and 51 (67.1%) children were female. Of these 76 children, 20 children did not log in (n=10) or failed to complete the questionnaire for other reasons (n=10), resulting in questionnaire data from 56 children (Figure 2). For in-person interviews, 29 children were eligible because they were within the 3-month intervention period and lived in the Northern or Middle part of the Netherlands. We invited 19 children to participate in an in-person interview, of which 9 declined to participate because of personal circumstances (n=3), no interest (n=3), or no further reason (n=3). In addition, 3 parents of the children with low adherence were invited for a telephone interview. Interviews contained 13 children, of which 4 did not fill in the questionnaire. A total of 23 interviews were conducted, which lasted 19‐63 minutes for in-person interviews and 5‐11 minutes for telephone interviews. The in-person interviews included 10 children and 12 parents, of which 2 interviews were performed with both parents. The telephone interviews included 3 parents of the children with low adherence (Table 1).

**Table 1.** Characteristics of interviewed participants.

ID	Gender	Age (years)	Gender of parent	Age of parent (years)	Parental educational level^a^	Sessions (n)^b^	Favorite exercise
1	Girl	8	Female	41	High	10	The rainbow planet
2	Girl	11	Female	36	High	31	The favorite place
3	Girl	12	Female	42	Low	12	The slide
4	Girl	11	Female and male	40 (mother) 45 (father)	High (mother) High (father)	46	The favorite place
5	Girl	13	Female	50	Intermediate	14	Beach without worries
6	Boy	9	Female	44	High	29	The slide
7	Girl	7	Female	37	High	63	The slide
8	Boy	13	Female	43	Intermediate	46	Beach without worries
9	Girl	9	Female	38	High	22	The favorite place
10	Girl	8	Female and male	46 (mother) 44 (father)	High (mother) High (father)	55	Beach without worries
11^c^	Girl	10	Female	Unknown	Unknown	0	Unknown
12^c^	Boy	12	Female	Unknown	Unknown	1	Unknown
13^c^	Girl	10	Female	Unknown	Unknown	8	Unknown

a Educational level was considered low (primary and lower secondary), intermediate (secondary vocational), or high (Bachelor’s degree or higher).

b Number of sessions until the date of the interview.

c Telephone interviews were conducted with a parent only.

### Adherence to Hypnotherapy

Of the 66 children that did log into the website, 3 children did 1 exercise, 3 children did 2 exercises, 8 did 3 exercises, 8 did 4 exercises, and 44 children did all 5. Consequently, 52 of 76 (68.4%) children adequately adhered to the intervention. The children did on average 25.5 (SD 23.2) sessions (median 17, range 1‐89), and listened to more than one exercise in an average of 6.8 (SD 9.0) sessions (median 4, range 0‐43). Log data per exercise are described in Table 2.

**Table 2.** Log data per exercise.

	Total	Exercise 1: breathing and relaxation	Exercise 2^a^: The favorite place	Exercise 2^b^: The favorite place and rainbow	Exercise 3^a^: The rainbow planet	Exercise 3^b^: The air balloon	Exercise 4: Beach without worries	Exercise 5: The slide
Duration (min:s)	—^c^	13:00	14:18	18:00	16:50	11:47	14:29	14:30
Duration listened, (min:s), median (minimum-maximum^d^)	—	12:15 (00:05-26:30)	12:20 (00:05-28:40)	14:45 (00:05-22:15)	15:55 (00:05-34:20)	11:05 (00:05-13:40)	13:50 (00:05-31:00)	13:55 (00:05-36:50)
Children ever started (n)	66	66	50	11	46	10	51	51
Sessions per child, median (minimum-maximum)	17 (1‐89)	4 (1-30)	5 (0‐41)	2 (0‐19)	5 (0‐63)	3 (0‐21)	3.5 (0‐36)	3 (0‐48)

a Exercise in the version for children aged <12 years.

b Exercise in the version for children aged ≥12 years.

c Not applicable.

d Maximum duration is higher than the exercise’s length when children listened to the same exercise multiple times consecutively.

### Experiences From Children and Their Parents

#### Overview

From questionnaire data and interviews, we found 6 themes that capture experiences from the children and their parents: impression of the exercises, not for everyone, influence of perceived effect, integrating exercises in daily life, content and practicalities of the website, and customization to personal preferences. For every quote, corresponding IDs from Table 1 are listed.

#### Impression of the Exercises

We found varying impressions of the therapy, ranging from nice, fun, and easy to stupid, boring, and difficult.

*Then you have the feeling that you are really on a beach, and you can put all the feelings you don’t want into a sand castle, so I really like that*.

[Child 10]

*The exercises themselves are a bit boring, because you have to keep doing the same thing every day*.

[Child 2]

Most children felt relaxed during or after listening to an exercise and often had a positive association with a topic or liked the story in the exercises. The children enjoyed that they could choose what they wanted to see or do in their imagination.

And I really like the slide exercise because I like sliding a lot, and you can choose a color, and you can choose how fast you want to go.

[Child 1]

Although most of the children liked to visualize during the exercises, some of the children thought too little was happening. The children had conflicting ideas about exercises that required more physical activity such as stretching and relaxing muscles, or writing down colors and feelings.

*I find the exercises boring because you just have to listen. I would rather have something to do, like filling in the colors of the rainbow planet*.

[Child 1]

*I liked just listening instead of having to actually do things*.

[Child 5]

The children particularly enjoyed exercises in the beginning, when they were new and the children did not yet know what would happen. For some of the children, exercises became boring over time.

*Because I know the exercise very well I can’t really imagine anything different than what I do now when I listen to the exercise*.

[Child 3]

The other children enjoyed knowing the exercises well.

*Now I already know a few pieces by heart, so then I enjoy listening to them over and over again*.

[Child 4]

Each exercise was experienced differently with diverse positive and negative aspects. An overview of impressions per exercise is presented in Multimedia Appendix 3.

#### Not for Everyone

From interviews with the parents it emerged that they expected both boys and girls with FAP or IBS to be suitable for hypnotherapy. However, some of the parents were more skeptical about a child’s age or developmental level. Although children of all ages thought that the exercises were easy or somewhat childish, a few of the children noted that exercises were too difficult; they included difficult words or were spoken too fast. Some of the parents of children aged 7 or 8 years noted that their child was around the minimal age or level to remain focused for the entire exercise or understand why these exercises could help.

*If she is just a bit older maybe then it might be a bit more effective. That she would understand it a bit better, and be better able to recall things in certain situations*.

[Mother 7]

The parents also noted that certain characteristics could help the children in doing hypnotherapy successfully, such as being calm, creative, less rational, and having patience and high imaginary skills. The parents mentioned that energetic children might benefit most from relaxing but might need more time and practice. Indeed, some of the children did not like that they had to sit or lay still.

*Because then I start thinking, shall I sneak and read something or just keep my eyes open, because I’m bored. I can’t sit still. Look, I’m always fidgeting with my fingers*.

[Child 1]

Some of the children needed time to become used to the exercises and understand how to follow instructions. A few of the children were never able to surrender to the exercises because, for example, they could not imagine the suggestion, they did not like the stories, or they felt something and could not give in to the feeling.

*My child often strongly resisted doing the exercises because they made her sleepy and she didn’t want to give in to that. “Because of them I can’t hear my own dreams anymore.”*

[Mother of girl aged 8 years, 24 sessions, reported in the questionnaire]

The parents mentioned that sometimes when their child was too energetic or too stressed from events or stimuli, they could not concentrate and do the exercises in order to relax.

*Now I had the idea that it even worked against her, that she first had to deal with her own things, and that hypnotherapy… that it wouldn’t go together*.

[Mother 1]

#### Influence of Perceived Effect

Feeling an effect of the therapy affected experiences of the children. Although a few of the children experienced less abdominal pain but did not like the exercises, most did. Primarily because it made their belly feel good, or because their pain decreased or disappeared. They also experienced other effects such as greater confidence, better sleep, more energy, more relaxation, a clearer mind, and more frequent school attendance.

*I really like it, because you know that it relaxes you a lot*.

[Child 3]

When the children experienced less abdominal pain either through the exercises or for another reason, some of the children and parents felt less need to continue the exercises and quit.

*When she was in pain and started an exercise, it helped to reduce the pain. But when she did not have pain, she did not feel like doing the exercises or see the benefit of them*.

[Mother 13]

Some of the children were not able to relax during the exercises, and felt that the exercises did not help to remove their pain. They felt stressed because it did not work for them. Doing hypnotherapy felt like an obligation; this resulted in resistance or discontinuation.

*We have decided not to do the exercises anymore because they led to more frustration than success*.

[Mother of girl aged 9 years, 9 sessions, reported in the questionnaire]

#### Integrating Exercises in Daily Life

Many of the children enjoyed doing the exercises on their own, without involvement of others who could distract them. A few of the children mentioned that they would not do hypnotherapy in the presence of friends, because they would not like to explain what it is, or be different from their friends or classmates. The other children sometimes listened together with their parents or siblings which made them feel more relaxed. The parents also played an important role by reminding their children to do the exercises, because they did not come up with it themselves, were not in the mood, or forgot it.

*Stimulate her to do the exercises. She doesn’t think to do them or ask for them. So I have to motivate her a bit*.

[Mother 1]

Most of the children listened to exercises before going to sleep. Taking a device to their room was sometimes difficult for the parents because they usually withhold their children from screen time before bedtime. The children and parents appreciated that the exercises were easily accessible for the children to listen at home where they felt comfortable and at ease, such as on their bed.

*I really like that you don’t have to go anywhere. That you can just sit or lie down in your own house, in your comfort zone*.

[Child 8]

Most of the children accomplished making it a routine in their daily schedule which made listening to the exercises easier and more fun.

*He definitely doesn’t want to skip it in the evening. It becomes a kind of evening ritual: tooth-brushing, pajamas on and listen, and then to bed*.

[Mother 8]

The parents appreciated being able to use this as a tool when necessary, such as when their child was stressed or energized and needed to relax. Not all of the children and parents managed to integrate hypnotherapy into their routine. Some of the children were put off by doing hypnotherapy again because of the time constraint. A few of the parents noted that there is not enough time in a day to add 15 minutes of hypnotherapy, because it was not feasible to do it before school or before bedtime.

*Because sometimes the exercises are long, you sometimes practice a bit less, or maybe even not at all*.

[Mother 9]

The parents from the children with low adherence mentioned that they never started hypnotherapy because the children and their parents could not find a good moment in the first place.

*Several times we thought of it, but then we thought, ah, we’ll do it later. And every time that later never came*.

[Mother 11]

#### Content and Practicalities of the Website

Information was perceived as clear and interesting, though there might have been somewhat too much information for younger children according to the parents. The distinction between exercises and explanation was not always clear on the website. The children thought that there was an extra exercise, but felt disappointed when they saw it was an explanation only.

Insight in log-in data (ie, a small red cross for not logging in, or a green check for logging in, both per day and weekly over the entire 3 months) was experienced both positively and negatively. For some of the children this insight motivated them to do the exercises and obtain more green checks, and for others this worked counterproductively; they felt frustrated, angry, or ashamed.

*If I haven’t practiced for a couple of weeks, for example, because I am busy, then I feel a bit, uh different, that I haven’t done it right, that there will be red crosses*.

[Child 3]

For most of the participants, the website functioned well technically. However, some of the children experienced that the exercise stopped when the screen automatically turned off. This got them out of their concentration and affected their experiences negatively.

*So then she’s totally caught up in the story, and then she has to turn all the way around, click on that thing and install herself again*.

[Mother 1]

The children preferred using different devices, such as a smartphone, tablet, or computer. Many of the participants noted that a website was unpractical, since the children needed to fill in their log-in codes every time, were dependent on a parent nearby, or were unable to do it on the go. Sometimes, this made doing the exercises a barrier for the children. The parents suggested the use of an app instead of a website.

*If he could just do it in an app, that you just use a password one time and then you can just turn it on, then it would have been easier and he would have done it more quickly*.

[Mother 6]

#### Customization to Personal Preferences

The first aspect that the children would have liked to customize is appearance of the website and the exercises. The children and parents would have preferred to choose from different voices which can differ in speed. Overall, the children liked the looks of the website, but they had different ideas: some liked it simple as it is, others would have liked brighter colors such as pink and purple. The children would have liked to choose between colors on the website, change the background, and add illustrations to the exercises.

*I thought that the colors could have been brighter. And just as with the iPad you can choose a background, I would like that you could also choose a background here. It really doesn’t look so nice, so you think, now, I’m going to do some ZelfHy exercises*.

[Child 1]

The second aspect that the children and parents would prefer to customize relates to the content. Most importantly, the participants would have liked more exercises. Only a few of the participants found 5 exercises sufficient. Animals, love, music, flowers, favorite animation, traveling, games, hobbies, and sports were recommended by the children as preferred topics to add more attractive exercises. According to the parents, topics should also be suitable for a child’s age or level to be more effective.

*Because I notice that not every exercise is suited for her. That you could better, yeah, make more personalized exercises. And with things going on in her own environment, because that is, of course, different for a 7-year-old than for a 17-year-old*.

[Mother 7]

Additionally, choosing from exercises with different duration, particularly exercises of shorter duration (eg, between 5 and 10 min) is expected to enhance adherence. Shorter exercises would allow children to do an exercise quickly during a weekday, and to combine more exercises in 1 session. In contrast, a few of the parents hypothesized that shorter exercises might yield less effect than long exercises. The participants noted that a combination of shorter and longer exercises would be nice. Longer exercises are deemed useful in the beginning, and once children understand how they work, shorter exercises are expected to keep children more focused and satisfied.

*I certainly think that the more often you practice, then I can imagine that at a certain point you want to go through such an exercise more quickly, because you have already formed a certain image of it, or you have given it certain colors, or you have associated a certain emotion with it. Then it doesn’t have to take so much time*.

[Mother 6]

Some of the participants noted that it would be more motivating if the website contained games, or if they could unlock new exercises. The parents also noted that a scoring or rewarding system could help to increase adherence.

*You could attach a kind of gamification to it, so that the child gets a certain reward when she has done it, so that she wants to do the exercises*.

[Father 4]

## Discussion

### Principal Results

Adherence to hypnotherapy varied greatly: some of the children never started hypnotherapy, some only logged in a handful of times, and most of the children adequately adhered to hypnotherapy by starting at least four exercises. The children experienced home-based guided hypnotherapy differently, both in general and per exercise. From interviews with the parents it emerged that hypnotherapy was sometimes difficult for those children who were young or had a low developmental level, could not sit still or surrender to the exercise, or were too energetic or stressed. Experiences were shaped by the influence of perceived effects, the ability to integrate exercises in daily life, and content and practicalities of the website. Ultimately, the children and parents would appreciate a therapy that can be customized to personal preferences for appearance and content.

### Comparison With Prior Work

This is the first study to evaluate experiences of children and their parents with home-based guided hypnotherapy, other studies evaluated other self-guided interventions for children with abdominal pain. A mixed methods study assessing an online self-guided intervention found that children aged 9‐15 years were satisfied with the intervention [22]. In our sample, we found more variation in the children’s experiences, ranging from boring to fun. Possible explanations for this disparity could be that we included a larger sample, also included children with low adherence, and that children with questionnaire data had a broader age range, namely 7‐17 years. Notably, it was a different intervention and this study showed that characteristics of the website or intervention also influence a child’s experiences. The mixed methods study found that children learned to cope with their pain through relaxation or distraction and felt better in general [22]. This is consistent with our findings that experiences of children were influenced by a perceived effect.

In our study, a long duration of the exercises was one of the reasons why everyday adherence was difficult. Some children easily integrated the exercises into daily life, but others had difficulties to make it a routine. The parents who motivated and stimulated their child to listen to an exercise were helpful in integrating exercises in daily life. A mixed methods study assessing a guided imagery app also found no consensus on preferable exercise duration when asking children and parents [23]. In contrast, another study assessing guided imagery found that sessions lasting 10 to 25 minutes were enjoyable, children needed no help or reminders from parents, and most children listened to the exercises more often than instructed [24]. Our results add to the knowledge that preferences for duration of exercises are dependent on children’s characteristics, ability to do the exercises, parental involvement and ability to integrate exercises in daily life. Home-based guided hypnotherapy that includes exercises with varying durations would allow that it is suitable and fitting for everyone, because it can be adjusted to children’s preferences and time available at that moment.

The children and parents liked the look of the website in general, and a few experienced small technical issues that negatively affected their experience. Another study also mentioned small technical issues in an online intervention [25]. Consistent with a previous study using an app, the log-in procedure was easy [23]. However, in our study, the log-in procedure was also perceived as inconvenient, because it was not easy to use on the go. The parents suggested that an app could solve this inconvenience. A study assessing desirable components in a digital management app for children with long-term, chronic conditions found no agreement on preferences for either a website that is suitable on multiple devices versus an app [26]. This is consistent with our results and suggests that an app is favored, but flexibility of use on other devices should also be taken into account.

This study amplifies what has been found before, that children enjoy being able to do an intervention at home. eHealth interventions can increase adherence to treatment and improve outcomes which might influence effectiveness of the therapy [20,27]. Between the children and parents, there are many individual preferences regarding number and duration of exercises, topics, voices, and looks. Additionally, the child’s age or level plays an important role. Consistent with prior literature, rewarding systems or gamification is important in eHealth interventions for children [23,28]. Our study emphasizes the importance of tailoring hypnotherapy to children’s age or developmental level, customizing it according to their personal preferences regarding appearance and content, and incorporating gamification components to enhance engagement.

### Strengths and Limitations

An important strength of this study is that we interviewed both children and their parents, with varying adherence ranging from zero adherence to daily adherence for 3 months. Interviews with the parents followed after interviews with children and allowed for more depth. Interviews were performed in their home environment, which allowed for a safe environment, and we therefore believe that we captured honest answers.

One limitation is that we primarily interviewed children and young adolescents (age range 7‐13 y). Therefore, our results might not be generalizable to adolescents. We aimed to interview adolescents aged 14 to 17 years old, but failed because very few adolescents participated in the RCT. Although questionnaire data did include adolescents, and we included an adolescent for member checking, more research is needed on experiences of hypnotherapy among adolescents. Another limitation is that we failed to interview more fathers, as they were not at home during the interview. We believe that this minority of fathers did not influence our study results, because the 2 included fathers did not introduce new themes.

### Clinical and Research Implications

This study highlights the importance of personalized home-based guided hypnotherapy to improve a child’s experience, and possibly to increase adherence. This is of essence in eHealth, where patients themselves are responsible for following the therapy. Hypnotherapy that is fun to do at home and fitted to each child might be easy to adhere to and more prompting for GPs to promote. Primary care might be a beneficial setting for home-based guided hypnotherapy, as GPs manage most children with these complaints. Providing a self-managing intervention in this setting might prevent referrals to pediatric care and reduce costs [29,30].

### Conclusions

The children’s and parents’ experiences varied greatly and were partly influenced by the topic or story in the exercise. For children who are young or have a low developmental level, cannot sit still, are unable to surrender to exercises, or are too energetic or stressed, home-based guided hypnotherapy might be difficult and needs optimization. Children liked hypnotherapy when they felt positive effects, could easily integrate the exercises in their daily life, or enjoyed the website’s content and usability. Children who did not feel effects or found exercises too long often disliked hypnotherapy. A website or an app that is easily accessible and contains short exercises could increase its use. Hypnotherapy that is adaptable to personal preferences on appearance and content could boost the experiences. In turn, positive experiences might lead to higher adherence, which potentially increases the effect of hypnotherapy and should be studied further.

## Supplementary material

10.2196/58301 Multimedia Appendix 1 Open-ended questions from the questionnaire.

10.2196/58301 Multimedia Appendix 2 Interview guides.

10.2196/58301 Multimedia Appendix 3 Impressions per exercise.

## Abbreviations

FAP: functional abdominal pain
GP: general practitioner
IBS: irritable bowel syndrome
RCT: randomized controlled trial
