# Supplementary material for: Children’s and Their Parents’ Experiences With Home-Based Guided Hypnotherapy: Qualitative Study
Source: JMIR Pediatr Parent. 2025 Jan 27;8:e58301. doi: 10.2196/58301 (PMC11789690; doi:10.2196/58301)
Supplement: Multimedia Appendix 1 [file pediatrics-v8-e58301-s001.docx]

**Questions asked for each exercise separately:**

1. On a scale from 0 to 100, what did you think of this exercise? A 0 means that you think this exercise is bad, and a 100 means that you think this exercise is excellent.
2. What things did you like about this exercise?
3. What things did you not like about this exercise?

**Questions in general:**

1. In general, what did you think of home-based guided hypnotherapy?
2. Which exercise did you most often listen to?
3. Why did you choose this exercise most often?
4. What things did you like?
5. What things would you change?
