# Supplementary material for: Children’s and Their Parents’ Experiences With Home-Based Guided Hypnotherapy: Qualitative Study
Source: JMIR Pediatr Parent. 2025 Jan 27;8:e58301. doi: 10.2196/58301 (PMC11789690; doi:10.2196/58301)
Supplement: Multimedia Appendix 2 [file pediatrics-v8-e58301-s002.docx]

**For children**

**Opening question**

- You participate in the ZelfHy study. Could you tell me how you feel about participating?
- Do you remember why you wanted to participate in the ZelfHy study?
- What were you hoping for?

**Information prior to the therapy**

Before you started, you had a short online meeting with one of the researchers. She gave information about functional abdominal pain and the exercises.

- What did you think of it?
- How did this help you?

**Logging in**

- How is logging in to the website going?
  - Do you do it alone?
  - Who helps you?

The first time you logged in, you saw information on the website about abdominal pain, the exercises en how the exercises may help you.

- What did you think of this?
- What did you think of the amount of information?
- How did this information help you?

**Website**

- What do you think of the website?
- What do you think about looks of the website?
  - Colors / patterns / buttons / pictures
- What would you change?
- Do you have suggestions to make the website better?
- Would you rather exercise in a different way? (e.g., using an app)
- *For older children:* Did you experience any technical issues?

**Practicing**

- When you are practicing, can you tell me what you do?
  - Where?
  - When?
  - How long?
- How do you practice?
  - Device
  - Have you tried practicing without using the website?
- Who helps you?
- *For older children:* What do you think of doing these exercises at home?
- In the beginning we asked you to practice as much as possible, preferably every day. How is that going?
  - What would make it easier to practice more?
  - Did you have a feeling of obligation, that you had to practice?

**The exercises**

Let’s go through every exercise. I have exercises with me on cards.

- Could you arrange the 5 exercises from most fun to least fun?
  - Why is this your favorite exercise?
  - Why is this your least favorite exercise?
  - Is this also the order in which the exercises helped you the most?

*Follow-up questions for every exercise:*

- - What do you think of this exercise?
  - How did this exercise help you?
  - What do you like?
  - What do you not like?
  - What was easy?
  - What was difficult?
- You may choose which exercise you do. Do you often choose the same exercise, or do you do a different one every day?
- Are you able to imagine what is mentioned in the exercise?
- If you could choose an exercise yourself, what would it be about?
  - Which other subjects would you like to listen to?
- What did you think of the order of the exercises?
- What do you think about the voice of the exercises?

**Book**

- You also received a small book. Do you use it?
- What do you think of it?
- How does this book help you?

**Looking back**

- Did the exercises help you?
- How do I, or others see that on you?
- What was that like before you did the exercises?
- Did you expect this?

**Closing question**

- What advice would you give other children with abdominal pain?

**For parents**

**Opening question**

- Your child participates in the ZelfHy study. Could you tell me how your child feels about participating?
- Why did you want your child to participate in the ZelfHy study?
- What were you hoping for?
- What changes did you expect to see with your child?

**Role as parent**

- When your child practices, what do you do?
  - Has this been the same from the start?
- How do you help your child?

**Information prior to the therapy**

Before your child started, you had a short online meeting with one of the researchers. She gave information about functional abdominal pain and the exercises.

- What did you think of it?
- How did this help your child?
- Did you miss any information?
- What did you think of the amount of information?

**Website**

- What do you think of the website?
- What do you think about looks of the website?
  - Colors / patterns / buttons / pictures
- What would you change?
- Do you have suggestions to make the website better?
- Would you rather exercise in a different way? (e.g., using an app)
- How is logging in to the website going?
- Did your child experience any technical issues?

**Practicing**

- When your child is practicing, what do you notice on him/her?
  - How is that when the exercise is finished?
- How does your child practice?
- What does your child tell you about the exercises?
- How did your child make the exercises their own?
  - Is your child able to use their imagination?
- What do you think of the exercises?
  - What would you change?
- In the beginning we asked your child to practice as much as possible, preferably every day. How is that going?
  - What would make it easier to practice more?
  - Did your child have a feeling of obligation, that he/she had to practice?
- Do you have negative experiences with the website or the exercises?

**Book**

- Does your child use the small book we sent?
- What do you think of it?
- How does this book help your child?
- What would you change?

**Looking back**

- What do you think of doing these exercises at home?
- Did your child need more help next to the online meeting and information provided?
- What did your child learn?
- What differences have you noticed with your child?
  - What was this like before?
  - Did you expect this?
  - How do you think this is possible?
- Do you have suggestions how we could improve the therapy?
- What is the next step?
- Do you expect that hypnotherapy is suitable for all children with functional abdominal pain?

**Closing question**

- What advice would you give other children with abdominal pain?
