# Supplementary material for: Children’s and Their Parents’ Experiences With Home-Based Guided Hypnotherapy: Qualitative Study
Source: JMIR Pediatr Parent. 2025 Jan 27;8:e58301. doi: 10.2196/58301 (PMC11789690; doi:10.2196/58301)
Supplement: Multimedia Appendix 3 [file pediatrics-v8-e58301-s003.docx]

| **Positive impression** | **Negative impression** |
| --- | --- |
| ***Exercise 1. Breathing and relaxation*** | |
| Doing something active | Difficult to fall asleep |
| Most effective exercise | Too much focus on body |
| Cloud is fun | Not using own’s fantasy |
|  | Cloud is boring |
|  | Becoming nauseous |
| ***Exercise 2. The favorite place^a^*** | |
| Most effective exercise | Difficult (words) |
| Cloud is fun | Too fast |
| Idea of having a safe place | Not using own’s fantasy |
|  | The countdown |
| ***Exercise 2. The favorite place + rainbow^b^*** | |
| Dreaming away | Thinking too much |
| Colors | Drinks (mentioned in exercise) |
|  | Too long |
| ***Exercise 3. The rainbow planet^a^*** | |
| (Fill in) colors | Having to fill in colors |
| Using much imagination | Weird |
| Appealing topic | Too long |
|  | Too much going on |
| ***Exercise 3. The air balloon^b^*** | |
| Not much thinking | Difficult to understand how it works |
| Another place |  |
| ***Exercise 4. Beach without worries*** | |
| Beach and sea sounds | Seagull sounds |
| Appealing topic | Difficult (words) |
| Most effective exercise | Countdown of the stairs |
| Using much imagination |  |
| ***Exercise 5. The slide*** | |
| Appealing topic | Feels like the gut |
| Sense of sliding | Infinite sliding |
| Easy to imagine |  |
| Using much imagination |  |

^a^Exercise in the version for children aged <12 years.

^b^Exercise in the version for children aged ≥12 years.
